# Adaptive Square-Shaped Trajectory-Based Service Location Protocol in Wireless Sensor Networks

**DOI:** 10.3390/s100504497

**Published:** 2010-04-30

**Authors:** Hwa-Jung Lim, Joa-Hyoung Lee, Heon-Guil Lee

**Affiliations:** Dept. of Computer Science and Engineering, Kangwon National University, Chuncheon Gangwondo, 200-701, Korea; E-Mails: jinnie4u@kangwon.ac.kr (J.-H.L.); hjlim@kangwon.ac.kr (H.-J.L.)

**Keywords:** service location, trajectory, data replication, load scalability, robust

## Abstract

In this paper we propose an adaptive square-shaped trajectory (ASST)-based service location method to ensure load scalability in wireless sensor networks. This first establishes a square-shaped trajectory over the nodes that surround a target point computed by the hash function and any user can access it, using the hash. Both the width and the size of the trajectory are dynamically adjustable, depending on the number of queries made to the service information on the trajectory. The number of sensor nodes on the trajectory varies in proportion to the changing trajectory shape, allowing high loads to be distributed around the hot spot area.

## 1. Introduction

Advances in wireless networking have set new paradigms in computing, including pervasive computing based on a large-scale wireless sensor network. A wireless sensor network, a type of *ad hoc* network (MANets), is designed to be an infrastructure-less, unattended, and rapidly-deployable network. A fundamental issue in wireless sensor network environments is the efficient location of the required service in the network. The service location protocol is imperative to the design of a wireless sensor network because each network node lacks prior knowledge of the service available in the network [1–7].

Service location in wireless sensor networks is a challenging problem for several reasons. First, due to a lack of infrastructure, there are no well-known servers in a pre-defined network structure. Second, energy scarcity in a network node in a wireless network necessitates the design of new service location protocols that are qualitatively different from those designed for the wired network. Third, in many cases, wireless networks may scale up to thousands of nodes, rendering the location problem even more challenging [8–20].

In pervasive computing, users receive information regarding the environment in real-time; therefore, the sensor network, which is the foundation of pervasive computing, should enable real-time access. In particular, service information is very time critical in pervasive computing; therefore, the service location protocol for the wireless sensor network should provide high accessibility to service information [21–23]. The easiest way to provide high accessibility is to periodically broadcast (flood) service information to the entire network. This method entails major energy consumption, but it is simple and some protocols use this approach.

To reduce the overhead associated with broadcasting, some protocols restrict the flooding area by forwarding packets in a specific direction, as cross shape or restricted regions. These schemes could reduce the broadcasting overhead but still require unnecessary replications if the service information is not popular. Load scalability is the ease with which a distributed system can expand and contract its resource pool to accommodate heavier or lighter loads; it is the ease with which a system or component can be modified, added, or removed to accommodate a changing load. Service location protocols should rapidly provide service information with a large number of users. Therefore, load scalability is an important metric for a service location protocol [24–40].

In this paper, we propose an adaptive square-shaped trajectory (ASST)-based service location method, which is a novel self-configuring, scalable, energy efficient, and robust service location protocol. ASST is based on Geographic Hash Table (GHT) and Trajectory Based Forwarding (TBF). GHT maps the geographic position of a sensor network field to a hash table. In GHT, the sensor node closest to the position where is computed by hash function is responsible for a set of key and data [6,8,9,11]. ASST stores service information in groups of sensor nodes, called a trajectory. A node wishing to publish (advertise) service information obtains a position through the hash function, and it then uses geographic-aided routing such as GPSR (Greedy Perimeter Stateless Routing) to store service information to the trajectory surrounding the hashed position, as in GHT [6,7]. ASST uses TBF to form a trajectory storing the service information. Replication between nodes in the trajectory reduces the network load on a node because queries from users are distributed to several nodes in the trajectory. To further distribute the network load, ASST adjusts the range and size of the trajectory in proportion to the frequency of user queries. In the next section, we review related work. Section 3 describes ASST, and Section 4 provides performance evaluation. We conclude the paper in Section 5.

## 2. Related Work

Conventional solutions related to this paper can be classified into the following two approaches: Data Storage architecture in a wireless sensor network and Service Location protocols in an *ad hoc* network, as shown in Table 1 [4,5,15,22,34,37,40].

In the early days of sensor networks, the data sensed by sensor nodes were collected by a base station and stored externally. Here, external storage means that the node providing the storage space is located separately from the sensor networks. For users on a wired network seeking to access and use the sensed data, there are no problems associated with the external storage. However, if users are mobile, it is difficult to access the external storage on the wired network. To address this problem, internal storage architectures are proposed. In internal storage, each sensor node saves the sensed data to its local storage. Users obtain the data by directly querying the sensor network. Internal storage architecture can reduce the data collection overhead; however, users have to query the entire network to find the data. Data-centric storage architecture provides fast data dissemination by storing the data on the basis of its name. Data-centric storage is an enhanced version of data-centric routing. A data-centric routing scheme presented firstly is directed diffusion. Directed diffusion uses flooding to advertise the interests from sinks to sources throughout the network [4,13]. GHT is a type of data-centric storage architecture. GHT is based on the Distributed Hash Table (DHT) that is the results of research efforts on peer-to-peer (P2P) computing networks. GHT was proposed for data-centric storage with geographic information in a sensor network. GHT is a geographic hash table system that hashes keys into geographic points and stores the key-value set at the sensor node closest to the hashed point. GHT uses geographic perimeter routing to identify a packet home node. GHT provides fast access to the data in the sensor nodes but does not take account of availability and scalability. In GHT, only the home node responds to user queries. This causes a concentration of network load and reduces the energy of the home node [6,22].

*Ad-hoc* networks and DHT share key characteristics in terms of self organization, decentralization, redundancy requirements, and limited infrastructure. However, node mobility and the continually changing physical topology pose a special challenge to scalability and the design of a DHT for mobile ad-hoc network. Using DHT over wireless sensor networks has gained a lot of attention in the research arena recently. In wireless sensor network, the most important issue in routing is to gather the routed information coming from sensor nodes to the sink node regardless of the identity of the donating node. The problem in this context is to locate efficiently the sensor node, which holds the data item with the minimum number of intermediate nodes to save network energy.

A ScatterPastry platform based on Pastry DHT as an overlay routing platform for distributed applications over wireless sensor network using Scatterweb nodes, a real-world wireless sensor platform was proposed in [27]. A topology-based distributed hash tables (T-DHT) as an infrastructure for data-centric storage, information processing, and routing in ad hoc and sensor networks was introduced in [28]. T-DHTs do not rely on location information and work even in the presence of voids in the network. Using a virtual coordinate system, we construct a distributed hash table which is strongly oriented to the underlying network topology. The mobile hash-table (MHT) [29] addresses this challenge by mapping a data item to a path through the environment. In contrast to existing DHT, MHT does not to maintain routing tables and thereby can be used in networks with highly dynamic topologies. Thus, in mobile environments it stores data items with low maintenance overhead on the moving nodes and allows the MHT to scale up to several ten thousands of nodes.

In [30], the appropriateness of using DHT routing paths for service placement in an SBON was evaluated, when aiming to minimize network usage. SBONs are one approach to implementing large-scale stream processing systems. A fundamental consideration in an SBON is that of service placement, which determines the physical location of in-network processing services or operators, in such a way that network resources are used efficiently. Service placement consists of two components: node discovery, which selects a candidate set of nodes on which services might be placed, and node selection, which chooses the particular node to host a service. By viewing the placement problem as the composition of these two processes we can trade-off quality and efficiency between them. For this, two DHT-based algorithms for node discovery, which use either the union or intersection of DHT routing paths in the Stream-based overlay networks (SBON), was considered and compared their performance to other techniques.

In [31] a GHT based service discovery protocol including the mechanism that constructs topology-aware overlay networks in wireless sensor network was proposed. It does not require a central lookup server and does not rely on multicast or flooding. A Similarity Search Algorithm (SSA) for efficiently processing similarity search queries was proposed in [32]. A data-centric storage structure based on the concept of Hilbert curve and DHT was presented and then an algorithm designed for efficiently probing the most similar data item for the sensor network was proposed. A dynamic geographic hash table for data-centric storage in wireless sensor networks was proposed in [33]. Unbalanced resource utilization problem was addressed by proposing a dynamic GHT solution that relies on two schemes—a temporal-based geographic hash table to achieve overall load balancing among sensor nodes over time and a location selection scheme based on node contribution potential to proactively adapt the system to network dynamics.

An effective hotspot storage management schemes to solve the hotspot storage problem in GHT was proposed in [35]. The scheme included the cover-up and multi-threshold mechanisms. The cover-up mechanism can adjust to another storage node dynamically when a storage node is full, while the multi-threshold mechanism can spread the data into several storage nodes for load balancing of the sensor nodes. Increasing Ray Search (IRS), an energy efficient and scalable search protocol, and k-IRS, an enhanced variant of IRS based on the GHT was proposed in [36]. The priority of IRS is energy efficiency and sacrifices latency whereas k-IRS is configurable in terms of energy-latency trade-off and this flexibility makes it applicable to varied application scenarios. The basic principle of these protocols is to route the search packet along a set of trajectories called rays that maximizes the likelihood of discovering the target information by consuming least amount of energy.

**Figure 1.**
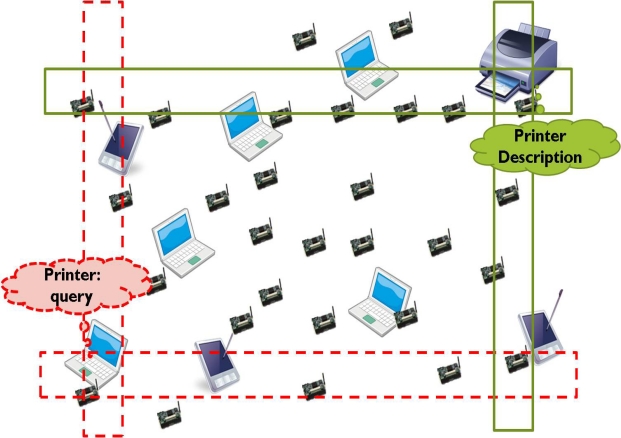
Service location in FMMS.

The classical protocols for service location in wired networks rely on a central server called a service directory. The central server advertises its information periodically. Users who want to use a service connect to the service directory in order to obtain a service description. In wired networks, the network topologies hardly change; therefore, users can access the service directory anytime. However, the central server cannot be used in a wireless network because the network topology in a wireless network frequently changes. The simplest form of service location is global flooding in the network; however, flooding does not scale well. To overcome the weakness of flooding, restricted flooding techniques are developed, such as the Facilitating Match-Making Service (FMMS) and Geography-based Content Location Protocol (GCLP) [5,15]. In FMMS, a service provider advertises the service in a cross-shaped trajectory along the network, as shown in Figure 1.

Service provider P sends a service advertisement packet in four directions, and the packet is forwarded until it reaches the boundary of the network forming the publish trajectory. A user C who wants to use the service also propagates the query packet in four directions, and the query packet is forwarded until it reaches the boundary of the network packet forming subscribe trajectory similar to advertisement trajectory. The nodes that belong to both trajectories (publish trajectory and subscribe trajectory) reply to user C with the service descriptions. GCLP reduces the query forwarding overhead by stopping the propagation of a query packet when the subscribe trajectory crosses the publish trajectory. FMMS and GCLP reduce the flooding overhead by limiting the flooding to four trajectories; however, both protocols are unable to provide scalability because they do not consider the query amounts, such that the trajectory always has the same size.

The distance-sensitive service discovery problem in wireless sensor and actor networks was formalized, and a novel localized algorithm, iMesh was proposed in [38]. Unlike existing solutions, iMesh uses no global computation and generates constant per-node storage load. In iMesh, new service providers (*i.e.*, actors) publish their location information in four directions, updating an information mesh such as GCLP. Information propagation for relatively remote services is restricted by a blocking rule, which also updates the mesh structure. Based on an extension rule, nodes along mesh edges may further advertise newly arrived relatively near service by backward distance-limited transmissions, replacing previously closer service location. The final information mesh is a planar structure constituted by the information propagation paths. It stores locations of all the service providers and serves as service directory. Service consumers (*i.e.*, sensors) conduct a lookup process restricted within their home mesh cells to discover nearby services. We analytically study the properties of iMesh including construction cost and distance sensitivity over a static network model.

A service discovery protocol based on hierarchical grid architecture in an ad hoc network which enhanced the GCLP was proposed in [39]. The geographical area was divided into a 2D logical hierarchical grid and the information of available services was registered to a specific location along a predefined trajectory. To enhance resource availability and effective discovery of GCLP, each grid cell selects a directory to cache available services. This work utilizes the transmitting trajectory to improve the efficiency of registration and discovery. First, the service provider registers a service along the proposed register trajectory. The requestor then discovers the service along the discovery trajectory to acquire the service information.

## 3. ASST

### 3.1. Basic Concept

**Figure 2.**
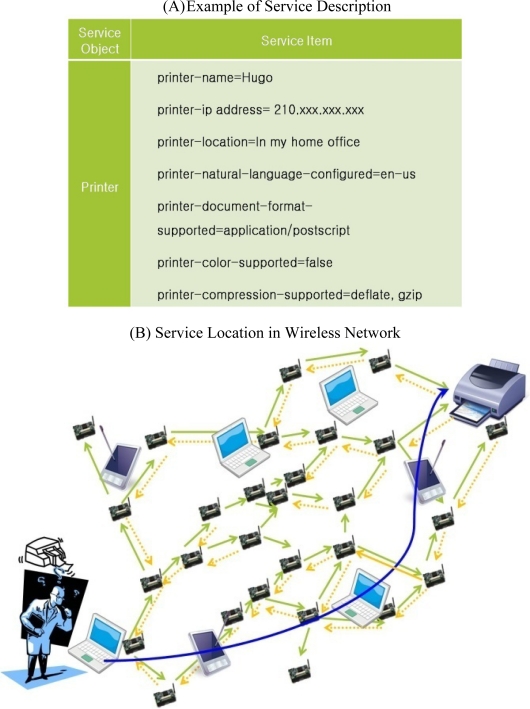
Example of service description and service location in a wireless network.

This section presents the basic design of ASST that is based on the following assumptions: (1) a vast field is covered by a large number of homogeneous sensor nodes that communicate with each other through short-range radios. (2) Each sensor node is aware of its own location and uses geographic routing such as GPSR to accomplish long distance delivery. (3) Service information means the service description that describes the characteristic of the service, as shown in Figure 2. (4) The storage space on the sensor node is sufficiently large to save the service information. Recently, sensor nodes with a very large memory space, such as RISE (RIverside SEnsor) with 1 GB of flash memory, have been developed so that sensor nodes can store a large amount of service information and occupy very little space [17].

Services have descriptions that include a service name and a service provider ID. Figure 2A shows an example of the service description of a printer. The service description can differ from applications to application and mainly depends on the types of services. Users wishing to use services provided by a wireless sensor network need to obtain the service description. The simple way to obtain the service description is by query flooding the entire network, as shown in Figure 2B. In the figure, a user with a laptop wants to print documents. Query packets with the service name that the user wants to use are broadcasted to all the nodes in the network until they reach the service provider matching the requirement in the query packet. The user connects to the printer server on the basis of the service description reply sent by the service provider. The flooding is easy and simple, but it may require a long time to discover the required service provider. Furthermore, the flooding could cause a considerable amount of energy consumption on the sensor nodes as a result of broadcast storming. Therefore, efficient service location protocols that provide service information quickly and with low network load are required in a wireless sensor network.

**Figure 3.**
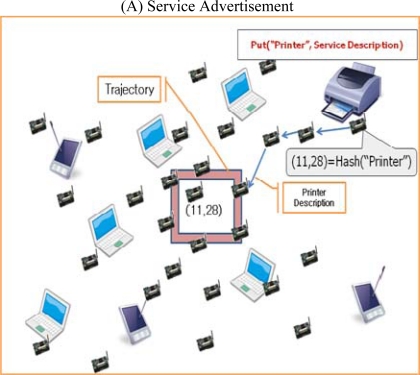

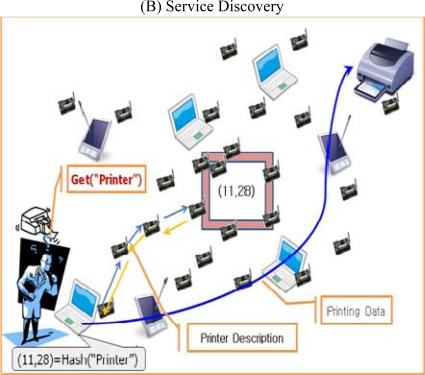
Example of ASST.

ASST is based on GHT and extends the DHT. Service descriptions that consist of a service name, a service provider ID, *etc.*, are stored in a distributed manner using an algorithm based on DHT. The specific service description is stored in repository nodes that correspond to the hash value of the service name. Geographic routing delivers the information for the service descriptions to and from the repository nodes. When a service provider wants to advertise its service description information on the network, the target point Q(T_x_, T_y_) hash key corresponding to the service name is first obtained using a well-known hash function such as MD5 and SHA. Once the target point Q(T_x_, T_y_) hash key is obtained, the service provider finds the repository nodes surrounding the hash value and stores the service description in those nodes. For example, in Figure 3, a node provides a printer service and wants to advertise its service to the network. First, the node finds the repository nodes for the printer by calculating the hash key for the printer service. If the printer’s hash key is (11, 28), nodes around (11, 28) become the repository nodes for the printer. A user wanting to use a printer finds the repository nodes for the printer by calculating the hash key of the printer service, same as the service provider.

ASST provides a general architecture for service discovery. Both the store and the query operations can be viewed as a general insertion and lookup, and the key can be looked-up in the hash table. Different applications can have different insertion and lookup characteristics. Therefore, different policies should be applied that are based on the characteristics of application. The popularity of data (equivalent to the data query frequency) is one of the most important characteristics of the lookup operation. Different data in an application may have different popularities, and this could lead to an unbalanced load distribution in the existing GHT.

**Figure 4.**
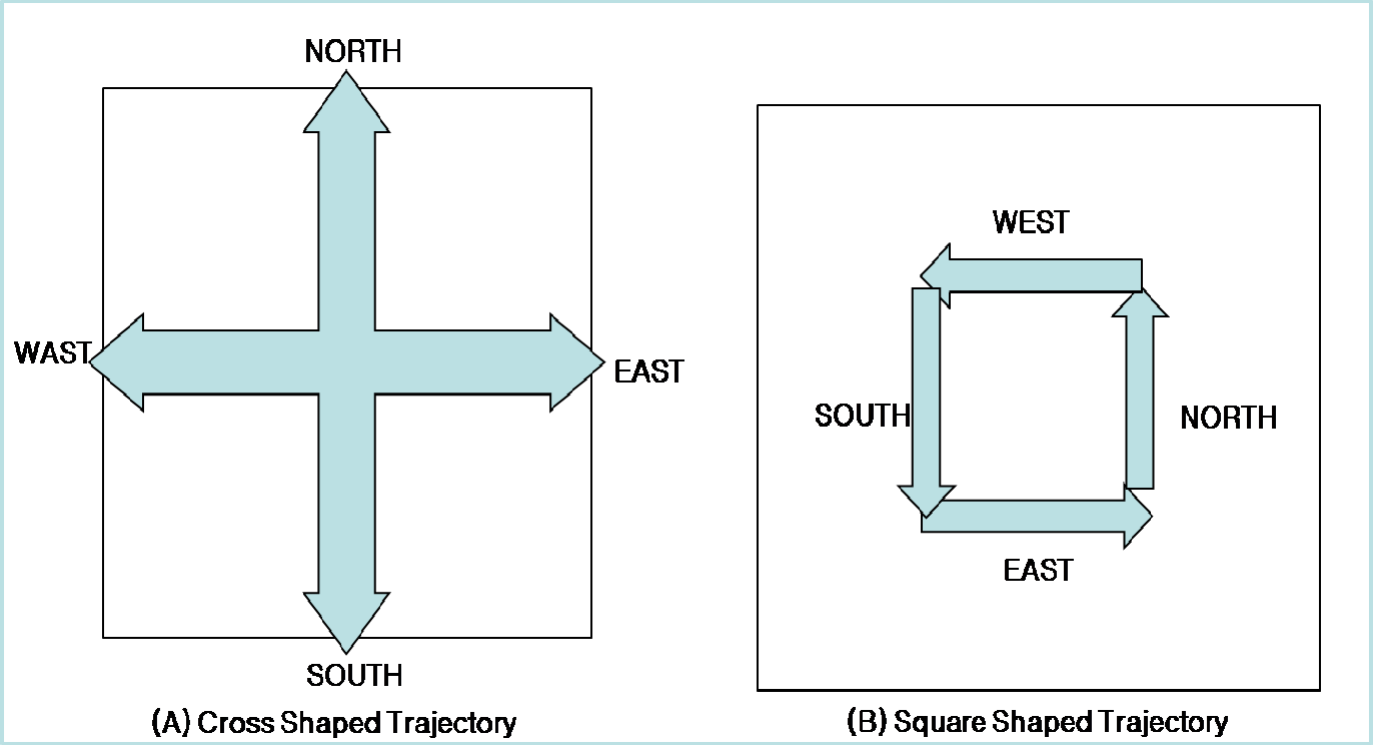
Cross Shaped Trajectory and Square Shaped Trajectory.

ASST transforms the cross shaped trajectory into the square shaped trajectory to control the number of nodes on the trajectory in proportion of popularity of data as shown in Figure 4. In the cross shaped trajectory, the publish trajectories are forwarded toward the boundary of sensor network field and the shape is fixed regardless of popularity of data. Therefore, it is possible some nodes on the trajectory not to receive any subscription trajectory (query packet) if the popularity if data is very low. Moreover, forwarding the publish/subscription trajectory toward the boundary of sensor network field can be overhead when a sensor network is deployed in large area. On the other hand, the shape of square trajectory can be increased or decreased easily and thus can be said more flexible than cross shaped trajectory. ASST distributes the query processing load to several replicated nodes, this is called trajectory zone. Trajectory zone in ASST is a square-shaped region with replica nodes for the data. A sensor node within the trajectory zone is called a repository node, which is responsible for the data storage and query response. To provide high scalability and accessibility for the data, ASST adjusts the range of the trajectory in proportion to the data’s popularity; this is referred to as dynamic trajectory.

### 3.2. Square-Shaped Trajectory

As in the case of many distributed hash table systems, ASST provides a *(key, value)*-based associative memory. Services are named with keys. Both the storage of the service and its retrieval are performed using these keys. Any naming scheme that distinguishes the services that users of the sensor network wish to distinctly identify will suffice in ASST [6]. ASST supports the following two operations:

**Put(***k; v***)** stores *v* (the observed data) according to the key *k*, the name of the service.
**Get(***k***)** retrieves whatever stored value is associated with key *k*.

**Figure 5.**
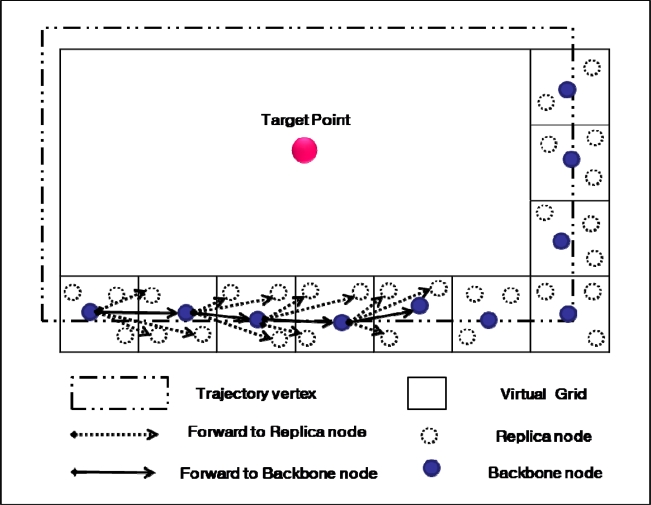
Trajectory formation.

First, the backbone node Rn in the trajectory Tr obtains data from the data packet sent by source S, and it then forwards the data packet to the next backbone node along edge E and toward vertex V in a counterclockwise direction, as shown in Figure 5. By overhearing the data packet forwarded by the backbone node, replica nodes around the backbone node obtain the data and store it. Source node S frequently sends data packets to update the data on the repository node. Each repository node keeps a timer for the data it stores. If the timer expires before any update packet is received from the source, then the data is discarded. To prevent query slippage in the trajectory, we use the method proposed in [9]. The method divides the network into a virtual grid and ensures that the trajectory is constructed through the sequential cells with sensor nodes. The grid line in Figure 5 shows the virtual grid in the network; the trajectory is formed by forwarding the data packet through the sequential cells. In [9], the trajectory was cross shaped; the trajectory in this paper is square shaped. However, the process of trajectory formation is the same for both, only the order differs.

**Figure 6.**
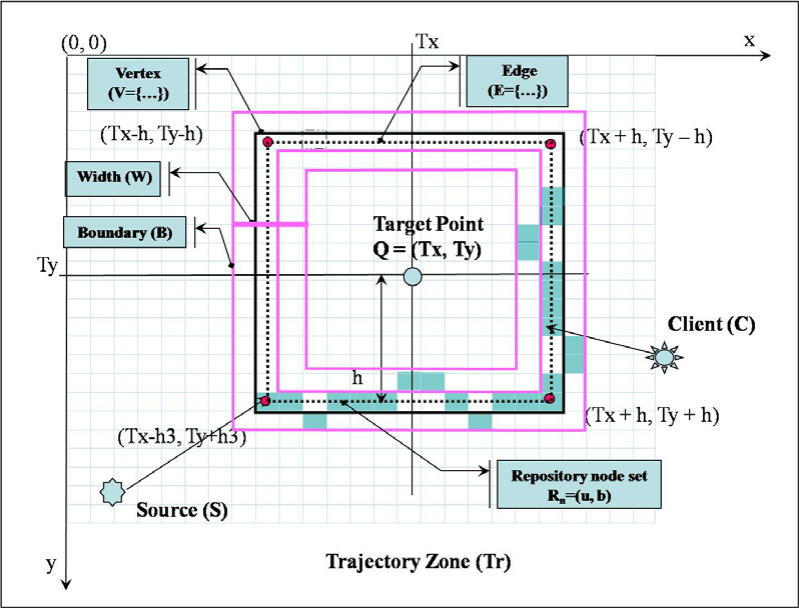
Square-Shaped Trajectory Formation.

Trajectory Tr is formed away from the target point Q with a distance H (h, h1, h2,… hn) computed in proportion to the data popularity (the same as the query frequency) p by the source node S. In ASST, source nodes only need to know the target point Q and the distance h to form the trajectory Tr. A node that receives a data packet from source S could check whether or not it is involved in trajectory Tr. Trajectory Tr is shaped with vertex V, edge E, and boundary B. Figure 6 shows the elements and formation process of the trajectory Tr. Vertex V of trajectory Tr is a corner of the square with a distance H(h, h1, h2,… hn) from the target point Q (Tx, Ty). The value of vertex V is one of (Tx − h, Ty − h), (Tx + h, Ty − h ), (Tx − h, Ty + h), and (Tx + h, Ty + h). Edge E of trajectory Tr is a line connecting two vertexes V. Trajectory Tr has a boundary B at each side of edge E with width W.

Trajectory Tr is a set of data-centric storage nodes, called repository node Rn. Repository node Rn consists of a backbone node b, which lies on the edge E of trajectory Tr, and replica nodes u, which lie between boundary B and edge E. Source S sends a data packet toward the target point. The header of the data packet contains the target point Q (Tx, Ty), range distance h, Source ID, and the Data ID. Each node between the source node and the target point computes the vertex V and edge E for the data packet. If the node is on the edge or vertex of the trajectory’s square, then the node becomes the backbone node and starts to form trajectory Tr.

### 3.3. Dynamic Trajectory

To provide scalability for a large sensor network, ASST dynamically adjusts the square range of the trajectory in proportion to the query frequency for the data. If the number of queries for the specific data increases, the range of trajectory Tr also increases. On the other hand, if the number of queries for the data decreases, the range of trajectory Tr decreases. This is known as Dynamic Trajectory.

To decide the distance H which is the range of trajectory Tr, ASST computes the data popularity (query frequency) p for the data. Initially, we gather the number of total queries on all repository nodes Rn. This information can be gathered through the summation of the number of queries at each node and by piggybacking this on the data packet. The repository node feeds back the query information by returning the data packet to the source node. First, the backbone node b in trajectory Tr gathers query counts from the replica nodes around it, and then it piggybacks the total query counts, which is a summation of the query counts, for both itself and the replica nodes. Next, the backbone node that receives the data packet forwarded from the previous backbone node, adds both its query counts and the replica node’s query counts to the query counts from the data packet, and then it forwards this to the next backbone node. When the data packet returns to the first backbone node, the data packet is returned to source S, which computes a new distance H.

**Figure 7.**
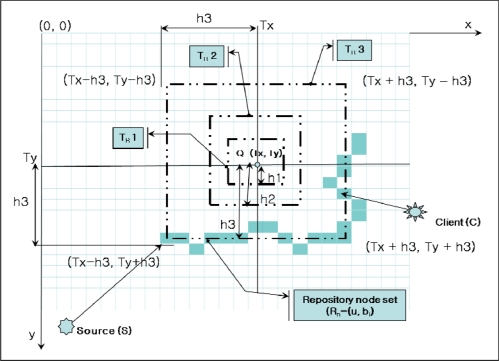
Dynamic Trajectory Development.

Figure 7 shows the Dynamic Trajectory in ASST. Dynamic Trajectory starts with a minimum distance h1. The source node S sends a data packet with a distance h1. At the next update, the source S sends a data packet with a distance h1 and computes a new distance with the query frequency. The new distance is not applied immediately but at the time of the next update. If the query frequency is increased over the threshold, the distance h is also increased to h2. At distance h2, if the query frequency is decreased, the distance is decreased to h1; if the query frequency is increased again, the distance becomes h3. When the distance is increased or decreased, a new trajectory is generated with the new repository nodes. We do not need to manually remove the old trajectory because each repository node has an update timer. If the data is not updated until the timer is expired, the node discards the data and this leads to deformation of the trajectory. Table 2 shows the procedures of dynamic trajectory in ASST.

### 3.4. Analysis

#### 3.4.1. Robustness

**Figure 8.**
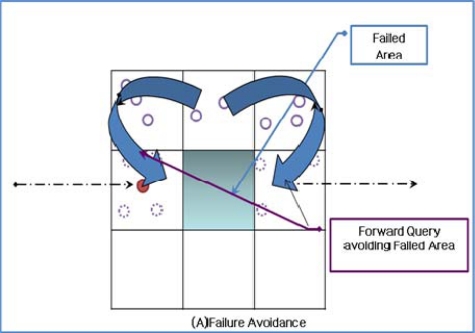

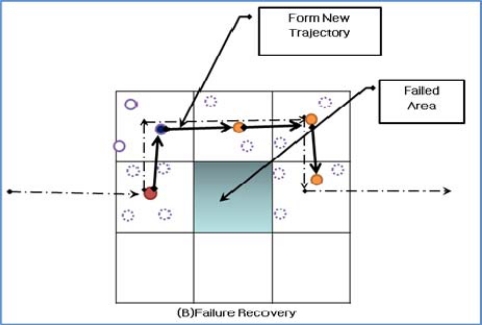
Failure Avoidance and Recovery.

Sensor networks consist of small fragile sensor nodes with limited resources such as computing power, energy, and network bandwidth. Sensor nodes can easily fail as a result of a physical attack or through energy dissipation. Therefore, sensor nodes should be protected, and systems running on sensor nodes should provide failure tolerance. The service location system should also be able to rapidly provide service information to the user, even if there are node failures in the network. ASST provides failure tolerance with a virtual grid when ASST forms a trajectory and the sensor node forwards the query packet, as shown in Figure 8.

A virtual grid on the trajectory consists of a backbone node and several replica nodes; other nodes in the virtual grid can respond to a query in the event of node failure, as long as at least one node is alive in the grid. If all the nodes in the grid fail, the query is forwarded to other grids by geographic routing, thus avoiding the failed area. Both the trajectory formation and query forwarding are based on the virtual grid; therefore, the query cannot miss the trajectory, as shown in [9]. When the service provider updates the trajectory, the trajectory is recovered by making a detour around the failed area. This technique can be extended for multiple grid failures.

#### 3.4.2. Load scalability

To be load scalable, a scheme has to offer uniform processing times irrespective of the loads. The service location protocol should be scalable to accommodate the variable popularity of a service. ASST increases the distance H of the trajectory in proportion to the query frequency in order to provide a uniform query processing time to the user. The number of virtual blocks on the trajectory with a distance H is ${\mathrm{NUM}}_{\mathrm{VB}}=\frac{4(2H)}{W}$, and the number of queries that one virtual block can process during T is ${\mathrm{NUM}}_{\mathrm{proc}}=\frac{T\times {\mathrm{SN}}_{\mathrm{Avg}}}{t_{\mathrm{proc}}}$, where t_proc_ is the processing time of one query on the sensor node and SN_Avg_ is the average number of sensor nodes in a virtual block. When the total number of queries during T is NUM_query_, the number of virtual block that requires to process the NUM_query_ is ${\mathrm{NUM}}_{\mathrm{reqVB}}=\frac{{\mathrm{NUM}}_{\mathrm{query}}}{{\mathrm{NUM}}_{\mathrm{proc}}}$ and thus the distance H_query_ is $H_{\mathrm{query}}=\frac{W\times {\mathrm{NUM}}_{\mathrm{query}}}{8\times {\mathrm{NUM}}_{\mathrm{proc}}}$. The required processing time for NUM_query_ is *t_req_* = *NUM_query_* × *t_proc_*. The query processing time per node is:

$$\begin{array}{l} t_{\mathrm{total}}=\frac{t_{\mathrm{req}}}{{\mathrm{SN}}_{\mathrm{total}}}=\frac{{\mathrm{NUM}}_{\mathrm{query}}\times t_{\mathrm{proc}}}{{\mathrm{SN}}_{\mathrm{Avg}}\times {\mathrm{NUM}}_{\mathrm{VB}}}=\frac{{\mathrm{NUM}}_{\mathrm{query}}\times t_{\mathrm{proc}}}{{\mathrm{SN}}_{\mathrm{Avg}}\times \frac{{8H}_{\mathrm{query}}}{W}}=\frac{{\mathrm{NUM}}_{\mathrm{query}}\times t_{\mathrm{proc}}}{{\mathrm{SN}}_{\mathrm{Avg}}\times \frac{8\times \frac{W\times {\mathrm{NUM}}_{\mathrm{query}}}{8\times {\mathrm{NUM}}_{\mathrm{proc}}}}{W}}\\ =\frac{{\mathrm{NUM}}_{\mathrm{query}}\times t_{\mathrm{proc}}}{{\mathrm{SN}}_{\mathrm{Avg}}\times \frac{{\mathrm{NUM}}_{\mathrm{query}}}{{\mathrm{NUM}}_{\mathrm{proc}}}}=\frac{{\mathrm{NUM}}_{\mathrm{query}}\times t_{\mathrm{proc}}}{{\mathrm{SN}}_{\mathrm{Avg}}\times \frac{{\mathrm{NUM}}_{\mathrm{query}}}{\frac{T\times {\mathrm{SN}}_{\mathrm{Avg}}}{t_{\mathrm{proc}}}}}=T \end{array}$$

Therefore, there is no delay time on the repository node.

#### 3.4.3. Time and message

For the data transmissions from a child sensor node to a parent node, CSMA is used to reserve the channel. We assume that each node knows the number of contending neighbor nodes (m) and contends the channel with the optimal probability p = 1/m. The probability that one contending node wins the channel is p_succ_ = (1 − 1/m)^m−1^. Since the number of slots needed until the successful reservation is a geometric random variable, the average number of contending slots (ACS) is given by:

$$\mathrm{ACS}=\frac{1}{{\left(1-\frac{1}{M}\right)}^{M-1}}$$

where M is the Average number of Neighbor nodes.

One node on the grid included to the Trajectory Zone has to forward the packet to next virtual block and therefore, the required time slots for Trajectory Zone (STZ) is given by:

$$\mathrm{STZ}=\mathrm{ACS}\times {\mathrm{NUM}}_{\mathrm{VB}}=\frac{1}{{\left(1-\frac{1}{M}\right)}^{M-1}}\times \frac{8H}{W}$$

The main part of procedure in Table 2 is finding the next node which is closest to the trajectory Edge and the time for this routine is in proportion to the number of nodes in a virtual zone, which is in proportion to the width of Trajectory Zone (W). Sorting with distance and finding a node minimum distance requires O(n log n) time. The required time for inter communication between backbone nodes is in proportion to the width of Trajectory Zone (W) and the distance H as shown above and thus can be also O(n log n). As a consequence, the total time complexity is O(n log n).

The number of message for Trajectory Zone formation is in proportion to the number of virtual block in Trajectory Zone since every backbone node in virtual blocks has to forward the formation message to next virtual block and thus the number of message for Trajectory Zone formation is given by O(N).

## 4. Performance Evaluation

In Section 3, we proposed ASST, a new mechanism for data dissemination based on DHT and TBF. In this section, we evaluate the performance of our proposed mechanism in ns-2 simulations. Ns-2 supports detailed simulation of mobile, wireless networks. Our simulation uses an 802.11 radio with a 30 m radio range, rather than the 250 m radio range of IEEE-compliant hardware; this choice is similar to that made in the evaluation of GHT with a 40 m radio range. ASST was implemented on the basis of GPSR. 900 sensor nodes were deployed in a 600 m × 600 m field in grid topology. The distance between sensor nodes was 20 m so that each sensor node has 8 neighboring nodes. The sensor nodes at the border line of the field were set as query nodes, frequently sending queries. The target point was set at the center of the field. Each sensor node consumes 0.5 w of power when it sends and 0.2 w of power when it receives. Table 3 provides details of the simulation parameters. RXThresh is a receive threshold, CPThresh capture threshold, and CSThresh carrier-sensing Threshold.

Scalability, as a property of systems, is generally difficult to define; however, it is essential to define the specific requirements for scalability based on the dimensions that are deemed important. In telecommunications and software engineering, scalability is a desirable property of a system, a network, or a process; it indicates the ease with which the system can either handle growing amounts of work, or can be readily enlarged. For example, it can refer to the capability of a system to increase total throughput under an increased load. An algorithm, design, networking protocol, program, or other system is said to be scalable if it is suitably efficient and practical when applied to large situations (e.g., a large input data set or a large number of participating nodes in the case of a distributed system). If the design fails when the quantity increases then it does not scale. In particular, load scalability means the ability of a distributed system to expand easily and to contract its resource pool to accommodate heavier or lighter loads. Alternatively, it is the ease with which a system or component can be modified, added, or removed to accommodate a changing load. In this paper, we use load scalability as an evaluation metric.

We evaluate the performance of ASST with a diverse query frequency. We compare ASST with GHT and GCLP. To evaluate the scalability of ASST, we varied the query frequency from 0.5 queries per second to 100 queries per second. To reflect query processing, each query has a query processing time of 100 ms. A replica node receiving the query packet has to wait for 100 ms before responding. To provide load scalability, each query has to maintain the response time of 100 ms. The query was uniformly distributed among sensor nodes.

**Figure 9.**
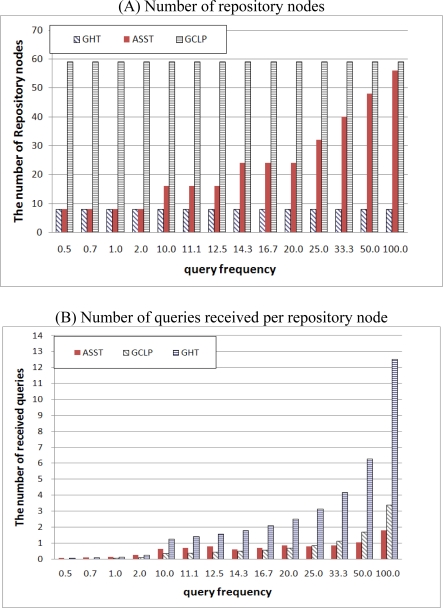
Number of repository nodes and the number of queries received per repository node.

Figure 9 shows the number of repository nodes and the number of queries received per repository node given several query frequencies. GHT saves information into very few repository nodes—home nodes and neighboring nodes surrounding the target point—and GCLP uses many repository nodes forming a trajectory from end-to-end in the network. Neither GHT nor GCLP take account of the query frequency; therefore, the number of repository nodes is fixed in spite of the increased query frequency. This leads to an increase in the number of queries received per node. On the other hand, ASST varies the size of the trajectory in proportion to the query frequency, so that the number of repository nodes in ASST is increased in proportion to the increased query frequency. Therefore, the number of received queries per node in ASST is smaller than that in GHT and GCLP. GHT can be regarded as minimum replication, and GCLP can be regarded as maximum replication. It should be noted that the number of repository nodes in GCLP and ASST is similar when the query frequency is high, but the number of received queries per node in GCLP is higher than that in ASST due to the two matching points in GCLP. In GCLP, both the publish trajectory and the subscribe trajectory form a cross, making two matching points. Therefore, the number of received queries is higher than that in ASST.

**Figure 10.**
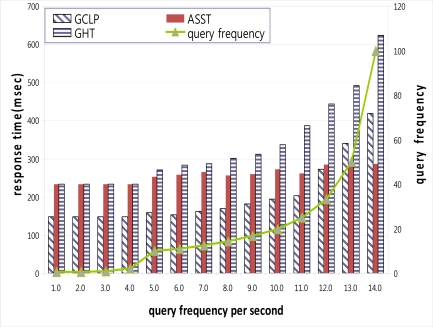
Response time with increased query frequency.

The number of queries per node can affect the response time. Figure 10 shows the response time with an increased query frequency. When the query frequency is low, GCLP shows a shorter response time than ASST or GHT because GCLP has more repository nodes than GHT or ASST. Moreover, GCLP forms a trajectory crossing the network so that GCLP occupies a larger area than GHT or ASST, and this leads to a reduction in the hop counts between the repository and query nodes. However, as the query frequency increases, the response time also increases. When the query frequency is very high, the response time is longer than that in ASST. This is caused by the larger number of received queries. However, GHT and ASST have the same number of repository nodes and the same hop counts between the repository and query nodes when the query frequency is low, so that the response times of both is the same. However, when the query frequency is increased, the response time of GHT is increased drastically while the response time of ASST is almost uniform. This is due to the difference between GHT and ASST in terms of the number of received queries per node. GHT always has a fixed number of repository nodes so that the number of received queries per node increases, and this leads to an increased response time. On the other hand, ASST increases the number of repository nodes in proportion to the query frequency so that the number of received queries per node and response time are almost uniform.

**Figure 11.**
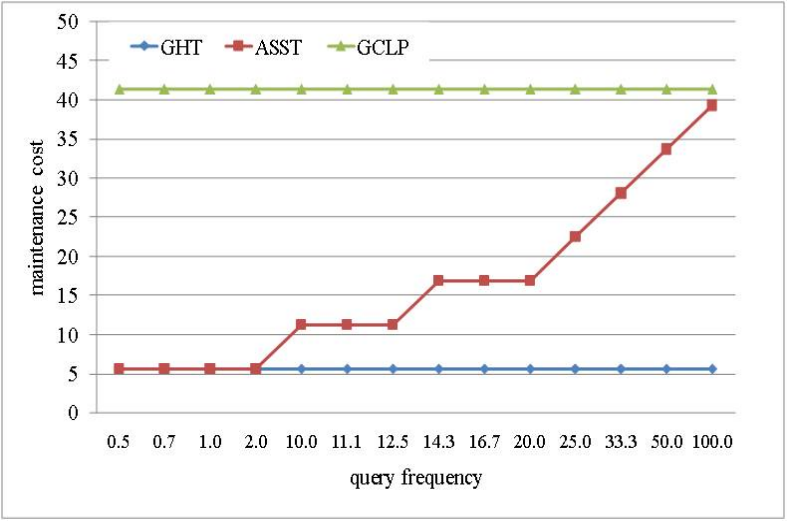
Energy consumption for storage construction.

Replicating data in several sensor nodes requires energy costs for packet transmission. More nodes participate in replication, and more energy is consumed because energy consumption is increased whenever a node transmits a packet. Figure 11 shows the energy consumption for repository construction. As mentioned earlier, the number of repository nodes in GHT and GCLP is fixed in spite of query frequency change. GHT maintains a minimum number of repository nodes around the target point so that the energy consumption is lower than that with GCLP or ASST. In contrast, GCLP maintains repository nodes between the network boundaries in a cross shape so that energy consumption is higher than that under GHT or ASST. On the other hand, ASST varies the number of repository nodes in proportion to the query frequency so that the energy consumption is low when the query frequency is low, and the energy consumption is increased in proportion to the query frequency. The energy consumption is similar with GHT when the query frequency is low, and the energy consumption is increased towards the GCLP as the query frequency increases.

## 5. Conclusions

In this paper, we have proposed a new energy efficient and scalable data dissemination method, *i.e.*, ASST, based on DHT and TBF. ASST is a type of data-centric storage system for sensor networks. ASST provides fast access to the data stored in a sensor network by using the distributed hash function. In ASST, the source node stores data at the nodes forming a trajectory around the target position computed by the hash function. A client sends a query packet to the position computed by the hash function that is the same as the source node. By storing data at several repository nodes, ASST distributes the network load caused by query packets.

ASST also provides scalability for a large sensor network. When the query frequency is increased, the range of the trajectory is also increased to reduce the network load at the repository node. By adjusting the range of the trajectory in proportion to the query frequency, ASST can ensure a fast response time for multiple clients and reduce the network load assigned to a sensor node. Such capabilities result in an increased life span for the sensor network.

The main focus of ASST is providing load scalability with constant response time by adjusting the width of trajectory in proportion to the query frequency under uniform distribution of query, however the query distribution might not be uniform over network and thus increasing or decreasing four edges with same distance H can cause load unbalance under non-uniform distribution of query. In the future work, we will consider the adjustment of distance H of four edges in trajectory zone in separated manner under with under non-uniform distribution of query.

## Figures and Tables

**Table 1.** Classification of Data Storage Schemes and Service Location Protocols.

	**Data Storage Scheme**	**Service Location Protocol**
**Wired Networks**	External Storage	Service Directory
**Wireless Networks**	Internal Storage	Flooding
	Data Centric Storage	FMMS
	GHT	GCLP

**Table 2.** Dynamic ASST procedure.

*procedure put*(*k*, *v*)
*TP*(*Tx*, *Ty*)← *Hash*(*k*)
*QF* ← *get* _ *Query* _ *Freq*(*k*)
*h* ← *QF* × *QT*^2^
*Vertex*[4] ← {(*Tx* − *h*, *Ty* − *h*), (*Tx* − *h*, *Ty* + *h*), (*Tx* + *h*, *Ty* + *h*), (*Tx* + *h*, *Ty* + *h*)}
*Min* _ *Dis* ← *MAX* _ *DIS*
*for i* = 1 *to* 4 *do*
*dis* ← *dis* tan *ce*(*my* _ *P*, *Vertex*[*i*])
*if dis* < *Min* _ *Dis then*
*Min* _ *dis* ← *dis*
*T* arg *et* _*Vertex* ← *Vertex*[*i*]
*Direction* ← *i*
*end if*
*end for*
*Send*(*US*, *k*, *v*, *TP*, *h*, *Vertex*, *T* arg *et* _*Vertex*, *Direction*, *ET*)
*return*

*procedure receiveVertex*(*k*, *v*, *TP*, *h*, *Vertex*, *T* arg *et* _*Vertex*, *Direction*, *ET*)
*if Us* = *my* _*US then*
*return*
*my* _*US* ← *US*
*my* _ *k* ← *k*
*my* _ *v* ← *v*
*my* _ *ET* ← *ET*
*set* _ *Timer*(*my* _ *ET*)
*if Direction* = *EAST then*
*Direction* ← *NORTH*
*else if Direction* = *NORTH then*
*Direction* ← *WEST*
*else if Direction* = *WEST then*
*Direction* ← *SOUTH*
*else if Direction* = *SOUTH then*
*Direction* ← *EAST*
*end if*
*T* arg *et* _*Vertex* ← *Vetext*[*Direction*]
*GN* ← *Grid* _ *Number*(*Direction*)
*Neighbor*[] ← *Neighbor* _ *Set*[*GN*]
*Backbone* ← min *dis*(*TP*, *h*, *Neighbor*)
*Send*(*US*, *k*, *v*, *TP*, *h*, *Vertex*, *T* arg *et* _*Vertex*, *Direction*, *Backbon*, *GN*)
*return*

*procedure receive* Re *pository*(*k*, *v*, *TP*, *h*, *Vertex*, *T* arg *et* _*Vertex*, *Direction*, *ET*, *Backbone*, *GN*)
*if US* = *my* _*US then*
*return*
*end if*
*if my* _ *GN* = *GN then*
*my* _*US* ← *US*
*my* _ *k* ← *k*
*my* _ *v* ← *v*
*myET* ← *ET*
*set* _*Timer*(*ET*)
*end if*
*if Backbone* = *my* _ *ID then*
*T* arg *et* _*Vertex* ← *Vertex*[*Direction*]
*Neighbor*[] ← *Neighbor* _ *Set*[*Direction*]
*Backbone* ←min *dis*(*TP*, *h*, *Neighbor*)
*end if*
*Send*(*US*, *k*, *v*, *TP*, *h*, *Vertex*, *T* arg *et* _*Vertex*, *Direction*, *Backbone*)
*return*

k : key of value

v : value

TP : Target Point

QF : Query Frequency

my_P : position of source

US : Update Sequence

ET : Expire Time

GN : Next Grid Number

**Table 3.** Simulation parameters.

**Parameter**		**Value**	**Parameter**			**Value**
Radio Propagation		Shadowing	Routing Protocol			GPSR
MAC		802.11	Energy Consumption		Idle Power	0.1 w
Queue		DropTail			Rx Power	0.2 w
Antenna		OmniAntenna			Tx Power	0.5 w
Network size		600 m × 600 m	Nodes			900 nodes
Radio Parameter	CPThresh	10.0	Packet size			60 bytes
	CSThresh	1.559e–11	Antenna Parameter	X		0
	RXThresh	3.652e–10		Y		0
	Pt	2.5872e–4		Z		1.5
